# Exploring the Origins of Low-Temperature Thermochromism in Polydiacetylenes

**DOI:** 10.3390/polym16202856

**Published:** 2024-10-10

**Authors:** Magdalena Wilk-Kozubek, Bartłomiej Potaniec, Patrycja Gazińska, Joanna Cybińska

**Affiliations:** 1 Materials Science and Engineering Center, Łukasiewicz Research Network—PORT Polish Center for Technology Development, 147 Stabłowicka Street, 54-066 Wrocław, Poland; magdalena.wilk-kozubek@port.lukasiewicz.gov.pl (M.W.-K.); bartlomiej.potaniec@port.lukasiewicz.gov.pl (B.P.); 2 Center for Population Diagnostics, Łukasiewicz Research Network—PORT Polish Center for Technology Development, 147 Stabłowicka Street, 54-066 Wrocław, Poland; patrycja.gazinska@port.lukasiewicz.gov.pl; 3 Faculty of Chemistry, University of Wrocław, 14 F. Joliot-Curie Street, 50-383 Wrocław, Poland

**Keywords:** low-temperature thermochromism, irreversible thermochromism, temperature indicators, thermochromic sensors, polydiacetylenes, side-chain length, hydrogen-bonding interactions, copolymers, biobanking, freshness sensors

## Abstract

This review article delves into the intriguing phenomenon of low-temperature thermochromism, whereby materials change color in response to temperature variations, with a particular focus on its applications in temperature-sensitive fields like medical storage. By closely examining thermochromic materials, this article highlights their potential to offer innovative solutions for monitoring and preserving thermolabile products that require strict temperature control. This leads to a special emphasis on polydiacetylenes (PDAs), a class of conjugated polymers with unique low-temperature thermochromic properties, positioning them as promising candidates for reliable temperature indicators. This article then explores the underlying mechanisms for fine-tuning the thermochromic behavior of PDAs, particularly discussing recent advancements in PDA design, such as structural alterations of monomers to achieve low-temperature thermochromism. These modifications, influenced by factors like side-chain length, hydrogen-bonding interactions, and the use of copolymers, are intended to result in irreversible color transitions at specific low temperatures, which is crucial to maintaining the integrity of thermally sensitive products. Finally, this article discusses the potential applications of PDAs as thermochromic sensors in tissue biobanking, where their ability to provide visual indications of temperature fluctuations could significantly enhance the monitoring and management of biological samples.

## 1. Introduction

**Figure 1:**
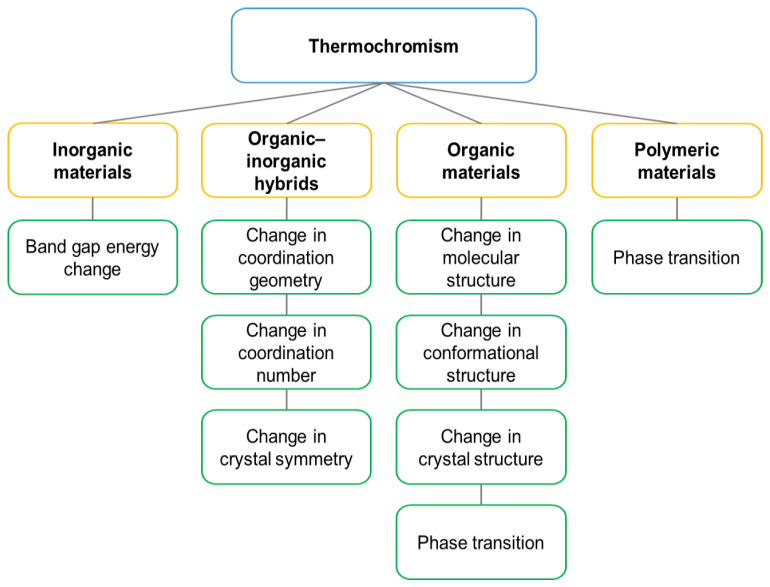
Mechanisms of action of thermochromic materials.

The phenomenon of thermochromism, characterized by the alteration in substances’ color in response to temperature changes, has fascinated scientists for centuries [1]. This process, which can be either reversible or irreversible, is driven by various mechanisms, such as molecular (conformational) and crystal structure (symmetry) transformations, alterations in coordination geometry and number, band gap energy shifts, and phase transitions (Figure 1) [2]. These mechanisms are observed in a wide range of materials, including inorganic [3], organic [4], organic–inorganic hybrid [5], and polymeric [6] ones. Due to their thermochromic behavior, these compounds hold significant potential for practical applications, inspiring innovative and environmentally friendly technologies. They form the basis of intriguing products, like invisible ink [7], heat-sensitive fax paper [8], and the iconic Hypercolor T-shirts [9]. Primarily, however, thermochromic materials that demonstrate color changes above ambient temperature serve as temperature indicators, providing crucial information about temperature variations [10]. For instance, they can measure temperature distribution in industrial heating devices [11], indicate temperature fluctuations in fire-resistant coatings [12], and monitor body temperature [13] or blood circulation [14] in medical applications (Table 1).

Recently, there has been a significant surge in interest in compounds exhibiting color alterations at sub-ambient temperature. This trend is primarily fueled by the increasing demand for advanced materials suitable for temperature sensors, which are essential to monitoring temperature-sensitive products like food, pharmaceuticals, and biological materials. These goods require strict temperature control during storage and transportation, as any deviations from optimal storage conditions can reduce their effectiveness [15,16]. For example, dairy products must be kept at 4 °C [17], while meat, fish, fruits, and vegetables need to be stored at −18 °C [18,19,20], and ice cream and confectionery products should be maintained at −29 °C [21]. In contrast, inactivated vaccines, dispersed cells, and polypeptide-based drugs such as insulin and antibiotics demand storage within a temperature range of 2 to 8 °C for short-term preservation [22,23,24]. Certain pharmaceuticals, like botulinum toxin A and methyl carprost, must be stored below −5 °C [25], while DNA and urine samples need to be kept at −20 °C for long-term preservation [26,27]. Moreover, live attenuated viral vaccines and red blood cells require storage for extended periods at even lower temperature, typically −70 and −80 °C, respectively [28,29].

Traditionally, the temperature monitoring of these thermolabile products has relied on temperature data loggers. However, the use of disposable models proves economically inefficient, while the retrieval and reuse of these devices present logistical challenges. Consequently, sensors employing thermochromic compounds emerge as a promising alternative, offering potential advancements in both cost effectiveness and logistical practicality for the monitoring of temperature-sensitive pharmaceuticals and biological materials [30]. In particular, compounds that undergo an irreversible color shift are more desirable for this application, as a color transition that cannot be reversed upon cooling provides a permanent record, indicating whether the products were exposed to excessive temperature at any point in the past. Moreover, the exact temperature at which the color change occurs is crucial, as it must align precisely with the temperature thresholds relevant to the stability of thermally sensitive products. While various materials with thermochromic properties can manifest irreversible color alterations, the greatest challenge is finding those that exhibit such transitions at specific low temperatures. For instance, inorganic and hybrid compounds generally do not reveal color shifts at sub-ambient temperature; instead, they change color at higher temperatures, ranging from 70 to 500 °C [31]. In contrast, organic materials, like leuco dye–developer systems, can alter color even at temperatures as low as −100 °C [32]. However, leuco dyes are typically sensitive to external factors such as moisture, oxygen, and pH changes, so they require protection [33]. This is achieved through encapsulation, which involves enclosing the dyes in protective coatings [34]. This method ensures better control over the conditions to which these organic compounds are exposed, regulates their release within strictly defined temperature ranges, and thus enables precise management of color shifts when mixed with developers [35]. Unlike organic materials, which commonly need a combination of active ingredients to attain thermochromic effects, polymeric materials inherently have the ability to undergo color transitions in response to temperature variations without requiring additional compounds [36]. This intrinsic capability arises from the unique structural properties of polymers, which can be engineered to exhibit thermochromic behavior over a broad temperature range, including sub-ambient levels, through direct modifications of their monomers. Consequently, polymeric materials offer a reliable and straightforward solution for managing temperature-induced changes, making them particularly valuable in applications necessitating accurate temperature monitoring.

**Scheme 1:**
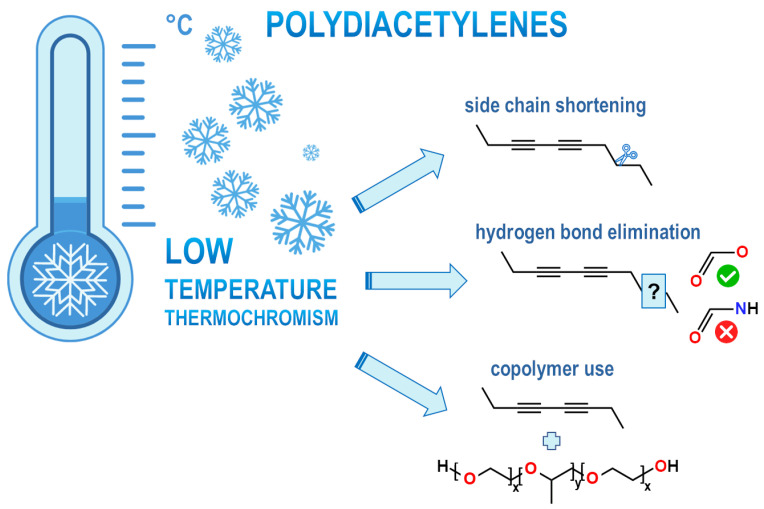
A general scheme showing the influence of the structural modifications of diacetylene monomers on the low-temperature thermochromism of the corresponding polydiacetylenes.

This review, therefore, focuses on the use of thermochromic materials, including polymeric ones, in the medical field, with special emphasis on biobanking. In this area, precise temperature control of storage conditions is crucial to preventing the degradation of thermolabile biological samples and ensuring their integrity for future research and medical applications. To meet these requirements, the compounds used need to demonstrate irreversible color-changing properties at sub-ambient temperature. Accordingly, this review highlights recent advancements in the design of polydiacetylenes, a subgroup of polymeric materials known for their low-temperature thermochromism. It explores innovative strategies involving the manipulation of monomer chemical structures, the interactions between monomers, and the integration of monomers into nanoscale architectures to fine-tune the thermochromic behavior of polydiacetylenes (Scheme 1).

## 2. Selection Criteria for Polydiacetylenes

Polydiacetylenes (PDAs) have been selected for low-temperature thermochromic applications, such as temperature indicators, due to unique properties that distinguish them from other materials. One of their key advantages is their ability, as single-component systems, to demonstrate a distinct color change—typically from blue to red—in response to temperature fluctuations [37]. This inherent characteristic allows PDAs to function effectively without the additional components, such as developers, required in systems based on leuco dyes. Consequently, the absence of these additives simplifies the design process and enhances the convenience of PDAs for practical applications. Additionally, the color alteration in PDAs is often more noticeable compared with leuco dye-based systems, where the change can be less pronounced and more gradual, depending on the strength of the developer. In contrast, color shifts in liquid crystals are angle-dependent, complicating interpretation and making them less effective for visual monitoring. Thus, the vivid color transition observed in PDAs makes them particularly suitable for visual temperature monitoring. Their high sensitivity to even slight temperature variations further underscores their appropriateness for precise temperature sensing. While leuco dyes generally exhibit lower sensitivity and are better suited for applications requiring a wider temperature range, they are not ideal for precise measurements. Although liquid crystals show high sensitivity, their optical changes are dependent on the angle of observation, limiting their usability in precision applications.

Moreover, PDAs can be easily chemically modified, allowing researchers to tailor their thermochromic properties to meet specific requirements. By introducing different functional groups, it is possible to fine-tune the temperature sensitivity, the range over which the color change occurs, and the intensity of this change, thereby customizing PDAs for various applications. In contrast, the ability to modify thermochromic properties in systems containing leuco dyes and liquid crystals is limited [38]. PDAs also offer the advantage of easy integration into various matrices, such as polymer films or membranes, thereby broadening their potential applications. Additionally, some PDAs can be designed to be more environmentally friendly than other thermochromic materials. For instance, organic–inorganic hybrids often contain hazardous heavy metals like lead [39], while leuco dye-based systems may include harmful components like bisphenol A [40]. In contrast, PDAs provide a safer alternative, making them an attractive option for applications where sustainability and environmental safety are critical considerations.

## 3. PDAs

PDAs are a class of conjugated polymers characterized by alternating double and triple bonds along their backbone. This ene-yne arrangement endows PDAs with thermochromic properties, making them a significant interest for various advanced applications, including temperature sensors [41], smart coatings [42], and biomedical devices [43]. First prepared by Wegner in 1969 [44], they are typically synthesized through a topochemical polymerization process. This solid-state method allows for precise control over the polymer’s molecular structure and eliminates the need for solvents, initiators, or catalysts [45]. Consequently, it minimizes side reactions and by-products, leading to the formation of high-purity PDAs without extensive post-synthesis purification steps [46].

**Scheme 2:**
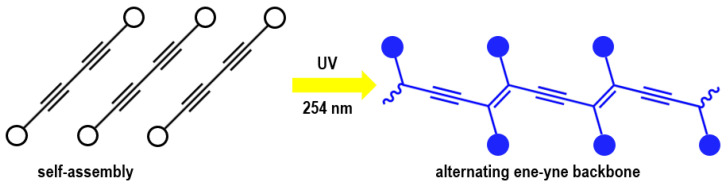
Topochemical photo-polymerization of DA monomer.

Efficient polymerization, however, requires diacetylene (DA) monomers to be pre-organized into highly ordered structures. For amphiphilic DA molecules, this organization usually occurs through self-assembly processes. Depending on specific conditions and the nature of the DA derivatives, self-assembly can lead to the formation of various architectures, such as micelles, vesicles, films, or monolayers [47]. In these self-assembled structures, the DA monomers are aligned approximately 5 Å apart, with each monomer oriented at about a 45° angle relative to the stacking axis of the assembly [48]. Upon exposure to ultraviolet (UV) light at a wavelength of 254 nm, the well-ordered DA derivatives undergo 1,4-addition polymerization (Scheme 2). This photochemical reaction leads to the formation of long conjugated polymer chains, which are characteristic of PDAs. PDAs typically undergo a gradual color change from blue to red upon heating. However, the detailed mechanism of the thermochromic transitions in PDAs is still under discussion. Earlier reports suggested a well-ordered planar structure for the blue state and a disordered non-planar structure with a reduced main-chain conjugation length for the red state. Recent reports, however, indicate that the thermochromic effect may originate from a transition between two different chain conformations, both of which may be perfectly ordered [49].

For better insight into the structural evolution of diacetylenes during photo-polymerization and subsequent heat treatment, a possible mechanism is proposed. Generally, DA monomers generate blue-colored polydiacetylene upon UV irradiation, which transforms to red when exposed to heat. The planar “blue” form has unbroken π-electron overlap and a full conjugation length. Upon heat treatment, the accumulated stress during photo-polymerization is released by the partial disordering of long alkyl chains, which eventually twist the π orbitals into non-planar states, reducing the effective conjugation length to that of the red form.

Temperature-induced conformational changes within the polymer chain result in shifts in the energy of the frontier orbitals, which are observed as visible color alterations and variations in the UV–vis spectra. Specifically, these changes lead to a blue shift (hypsochromic effect) in the absorption bands of the UV–vis spectra. The blue PDA phase typically shows an absorption band centered around ~640 nm, due to π-electron delocalization along the conjugated polymer backbone. As the temperature increases, structural alterations shorten the conjugation length of the polymer chain and increase the energy gap. This results in a shift of the absorption band to ~540 nm, indicating the transition to the red PDA phase [46]. In addition to the commonly observed blue and red phases, other chromatic states, like purple and orange, can also occur, with absorption bands at ~590 and ~530 nm, respectively. The presence of these states allows for the observation of a purple-to-orange phase transition upon heating. Further temperature increases can enhance the hypsochromic effect, leading to the yellow PDA phase, characterized by an absorption band around ~470 nm [50].

The color changes discussed above can be either reversible or irreversible, depending on the chemical structure of the PDA and the nature of the interactions between its chains. Specifically, π–π stacking interactions, hydrogen bonding [51], and other non-covalent interactions between the chains play a crucial role in determining this behavior. These interactions cause the chains to arrange closely, which influences the conjugation of the π-electron system along the chains. Consequently, when PDAs with strong intermolecular interactions are exposed to elevated temperatures, their chains undergo minor conformational changes that alter the torsion angles without permanently breaking chemical bonds. These slight modifications in chain arrangement affect the length of the conjugated system, leading to a transition in the observed color. Since these alterations are minor, the PDA can revert to its original conformation and color once the temperature is lowered, demonstrating the influence of chemical interactions on thermal response and color reversibility. Conversely, when the intermolecular interactions are weak or absent, significant structural changes and alterations in the conjugated system occur during heating, resulting in irreversible color shifts [52]. In such cases, the PDA does not return to its original state upon cooling, as the new conformation represents a permanent alteration that is not easily reversed. Thus, the nature of the intermolecular interactions between the PDA chains and the extent of structural modifications in response to temperature fluctuations significantly impact whether color changes are reversible or permanent.

Understanding the aspects that influence whether color changes in PDAs are reversible or irreversible is crucial, as is determining the temperature at which these changes occur. This temperature, also known as the thermochromic transition temperature, is influenced by the chemical structure of the PDA, including chain length, types of functional groups, and strength of non-covalent interactions between PDA chains [53,54]. These factors closely resemble those affecting the melting point of DA derivatives. As a result, the thermochromic transition temperature of PDAs often aligns closely with the melting point of the corresponding DA monomers [53]. This similarity arises because both thermochromism and melting are associated with overcoming energy barriers resulting from similar intermolecular interactions, thus requiring comparable temperature to induce these processes. Consequently, different strategies aimed at lowering the temperature needed for thermochromic transition involve making structural modifications to the DA monomer to effectively reduce its melting point. By doing so, the energy barrier related to these processes is lowered, allowing the color changes to occur at the temperature required for a specific application.

## 4. Influence of Side-Chain Length

Achieving low-temperature thermochromism in PDAs primarily involves designing DA monomers with short alkyl side chains. Such derivatives exhibit a notably lower melting point, consequently leading to a reduced thermochromic transition temperature compared with counterparts with longer alkyl side chains [55]. The phenomenon of decreasing melting point with shortening chain length, observed in DAs with alkyl side chains, resembles the trend seen in linear alkanes [56]. This similarity can be attributed to the reduction in chain length, which diminishes the energy associated with attractive van der Waals forces, thereby requiring less energy to overcome them. Therefore, this results in both a lower melting point and a reduced thermochromic temperature [57].

**Scheme 3:**
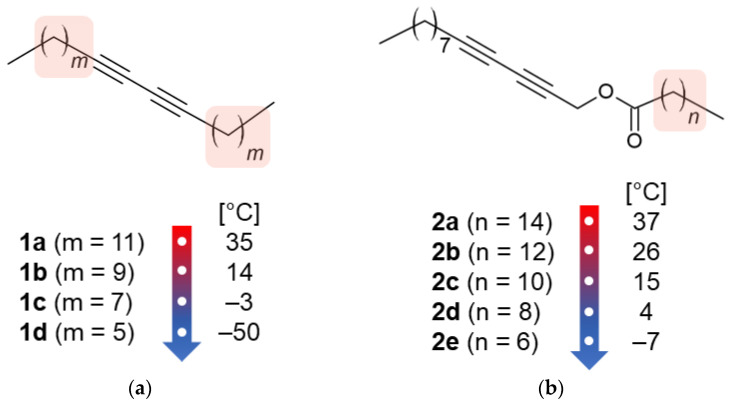
(**a**) Chemical structures of diyne monomers **1a**–**1d** and the thermochromic temperature of their polymers; (**b**) chemical structures of ester monomers **2a**–**2e** and the thermochromic temperature of their polymers.

Rougeau et al. demonstrated the efficacy of this approach by investigating a series of symmetric diynes and diyne esters containing alkyl groups [58]. In their study, diynes with longer alkyl chains, comprising 11 and 9 methylene units, exhibited the color transition at 35 and 14 °C, respectively. In contrast, diynes with shorter alkyl chains, featuring seven and five methylene groups, displayed the color shift at the significantly lower temperatures of −3 and −50 °C, respectively (Scheme 3a). These results clearly indicate that the thermochromic transition temperature decreases with a reduction in the number of carbon atoms in the side chains. However, it is worth noting that relying solely on chain length to predict the accurate temperature at which the color change will take place presents challenges, as there is not a strong correlation between these variables.

The situation becomes more straightforward when considering the thermochromic behavior of esters derived from trideca-2,4-diyn-1-ol and linear carboxylic acids [58]. In these esters, which contain 14, 12, 10, 8, and 6 methylene groups in the carboxylate moiety, the color change manifests at temperatures of 37, 26, 15, 4, and −7 °C, respectively (Scheme 3b). This observation suggests a more discernible relationship between chain length and thermochromic temperature. Specifically, for every decrease of two carbon atoms in the chain, the temperature drops by approximately 10 °C.

## 5. Influence of Methylene Groups Close to DA Moiety

**Scheme 4:**
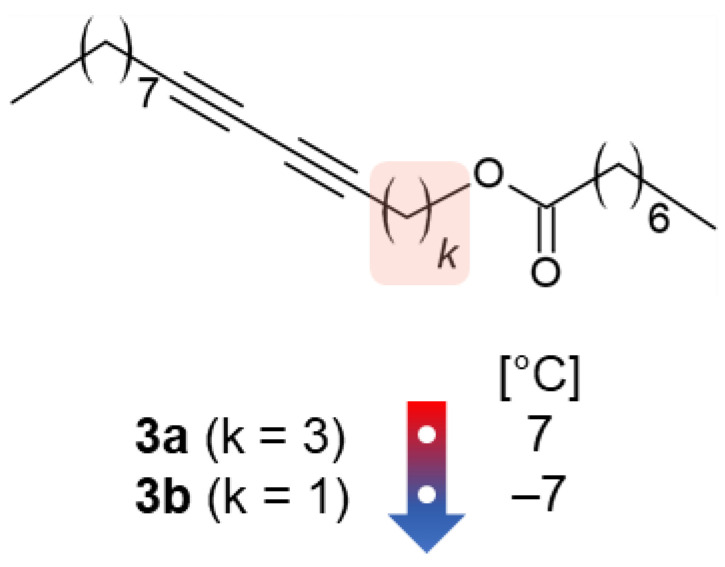
Chemical structures of ester monomers **3a**–**3b** and the thermochromic temperature of their polymers.

An alternative method for inducing low-temperature thermochromism in PDAs involves obtaining DA derivatives with the minimum number of methylene groups directly attached to the DA unit. The length of the linker connecting the DA portion to any functional group, such as an ester group, exerts a comparable influence on the temperature at which the color change appears, as does the length of the side chain. Rougeau et al. observed that introducing a three-methylene linker in the ester of pentadeca-4,6-diyn-1-ol results in the color transition at 7 °C (Scheme 4). Conversely, for the ester of trideca-2,4-diyn-1-ol, incorporating a methylene linker causes the color shift to occur at a lower temperature of −7 °C [58]. This finding suggests that reducing the length of the methylene linker contributes to a decrease in the thermochromic transition temperature.

## 6. Influence of Hydrogen-Bonding Interactions

A distinct approach to promote low-temperature thermochromism in PDAs involves designing DA monomers with functional groups that hinder the formation of strong interactions, particularly hydrogen bonds, among the polymer’s side chains. The presence of such functional groups, similarly to other organic compounds, leads to a reduction in attractive van der Waals forces, consequently lowering the melting point [59]. This reduction, in turn, contributes to a decrease in the thermochromic transition temperature of DA derivatives.

**Scheme 5:**
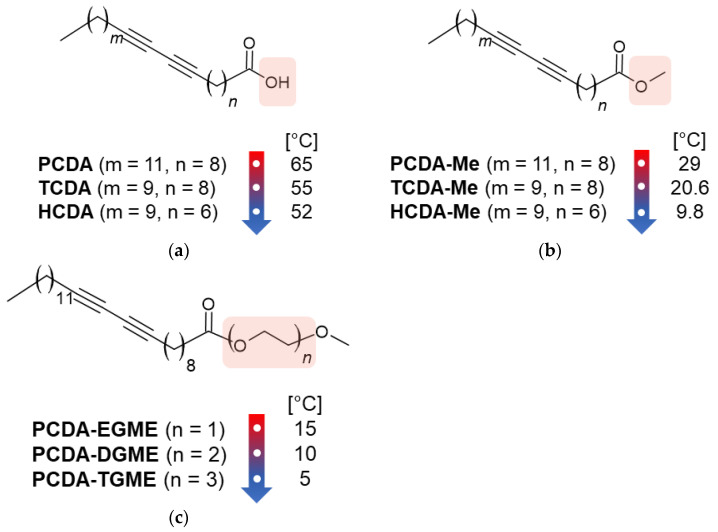
(**a**) Chemical structures of PCDA, TCDA, and HCDA monomers, along with the thermochromic temperature of their polymers; (**b**) chemical structures of PCDA-Me, TCDA-Me, and HCDA-Me monomers, along with the thermochromic temperature of their polymers; (**c**) chemical structures of PCDA-EGME, PCDA-DGME, and PCDA-TGME monomers, along with the thermochromic temperature of their polymers in PVA films.

The literature provides several examples that elucidate this concept, one of which involves the thermochromic behavior of poly-pentacosa-10,12-diynoic acid (poly-PCDA) and its ester derivatives. Park et al. observed that poly-PCDA demonstrates a color change at 65 °C [55] (Scheme 5a). Conversely, its methyl derivative (poly-PCDA-Me) exhibits a thermochromic transition at a lower temperature, approximately 29 °C [55] (Scheme 5b). For its esters with ethylene glycol monomethyl ether (EGME), diethylene glycol monomethyl ether (DGME), and triethylene glycol monomethyl ether (TGME), incorporated into a poly(vinyl alcohol) (PVA) polymer matrix, the color shift occurs at even lower temperatures. Specifically, poly(PCDA-EGME)/PVA, poly(PCDA-DGME)/PVA, and poly(PCDA-TGME)/PVA films manifest a color change at temperatures above 15, 10, and 5 °C, respectively [60] (Scheme 5c). The higher thermochromic temperature of poly-PCDA compared with its ester derivatives is ascribed to the presence of carboxyl groups, which facilitate the formation of hydrogen bonds. The replacement of acidic head groups with ester functional groups results in a lower transition temperature for the ester compounds because the potential hydrogen bonding between ester groups is either weak or absent. The notably lower thermochromic temperature of glycolic esters compared with poly-PCDA-Me is likely due to weaker attractive forces between ethylenoxy groups. Additionally, the reduction in transition temperature observed for glycolic esters with a higher number of ethylenoxy units may be attributed to increased strain in the side chains.

In their next study, Park et al. transformed the PCDA monomer into its isocyanate derivative, 1-isocyanatotetracosa-9,11-diyne (TCD-NCO), using a two-step process. Analysis of the thermochromic properties revealed that poly-TCD-NCO shows a color shift near 11 °C [61].

It can be noticed that the isocyanate derivative, similar to ester derivatives, exhibits a lower thermochromic temperature compared with poly-PCDA. It occurs because an isocyanate head group also lacks strong interaction capabilities.

**Scheme 6:**
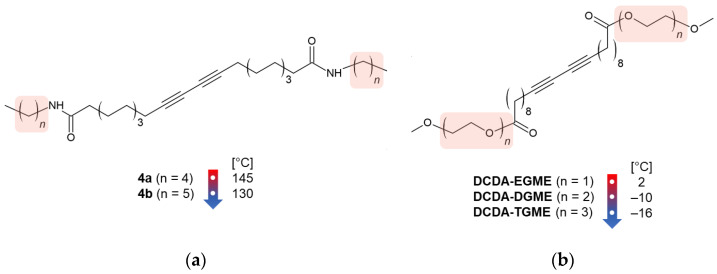
(**a**) Chemical structures of amide monomers **4a**–**4b** and the thermochromic temperature of their polymers; (**b**) chemical structures of DCDA-EGME, DCDA-DGME, and DCDA-TGME monomers, along with the thermochromic temperature of their polymers.

Another instance illustrating this strategy relates to the thermochromic behavior of diamide and diester derivatives of poly-docosa-10,12-diynedioic acid (poly-DCDA). Wrackmeyer et al. demonstrated that its diamides with *n*-pentyl and *n*-hexyl substituents on the termini undergo a color transition at 145 and 130 °C, respectively [62] (Scheme 6a). Meanwhile, Mergu and Son synthesized its diesters with EGME, DGME, and TGME, which manifest alteration in color at 2, −10, and −16 °C, respectively [50] (Scheme 6b). The thermochromic properties of poly-DCDA used to obtain these derivatives are not known. However, according to the generally accepted principle, it can be expected that poly-DCDA will reveal a color change at a temperature close to the melting point of the DCDA monomer (CAS: 28393-02-4), which is 110–112 °C. It is noteworthy that symmetric amides capable of forming hydrogen bonds show transition temperatures that are as high as that of poly-DCDA. They are significantly higher compared with those of symmetric esters, which lack this ability. Furthermore, despite the substantial difference in transition temperature between diamides and diesters, it is evident that in both cases, the temperature decreases with an increase in the number of methylene and ethylenoxy units in the side chains. This decrease may result from increased strain in the side chains.

## 7. Influence of Copolymer

Exploring low-temperature thermochromism in PDAs takes a completely different direction with the application of copolymers. This approach involves blending DA monomers with copolymers, resulting in nanoblends that effectively lower the chromatic transition temperature of PDAs while preserving the DA structure. This reduction in transition temperature is achieved by fine-tuning the hydrophobic/hydrophilic balance, average molecular weight (M_n_), and concentration of the copolymer.

A significant advantage of this method lies in its ability to utilize known DA derivatives, as presented in a study conducted by Ferreira et al., which examined the thermochromic properties of poly-PCDA and poly-tricosa-10,12-dyinoic acid (poly-TCDA) [63]. For comparison, the polymerization process was carried out by using classic vesicles, as well as special amphiphilic reactors formed by various triblock copolymers (TCs), including L35, L64, P123, F68, and F127. These copolymers, composed of ethylene oxide (EO) and propylene oxide (PO) segments arranged in an alternating EO-PO-EO linear fashion, vary in the number of EO and PO segments, as well as M_n_ [64].

**Scheme 7:**
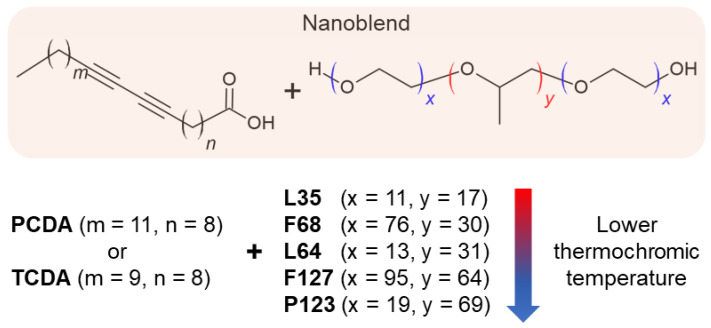
Chemical structures of PCDA and TCDA monomers and the thermochromic temperature of nanoblends of their polymers with the corresponding copolymers.

The study revealed that while poly-PCDA in vesicles exhibits a color change at 60 °C, incorporation of copolymers into nanoblends generally decreases the temperature of the chromatic transition. Additionally, increasing the concentration of the copolymer leads to a further reduction in the transition temperature. Notably, among the tested copolymers, the sequence L35 < F68 < L64 < F127 < P123, with an increasing number of PO segments (17 < 30 < 31 < 64 < 69), induces the most significant decrease in transition temperature (Scheme 7).

Of particular interest is the use of the P123 copolymer in poly-PCDA nanoblends, which triggers a color shift within a temperature range of 12 to 18 °C. This range is over 40 °C lower than that observed with poly-PCDA in vesicles. This observation suggests distinct intermolecular interactions of poly-PCDA in amphiphilic reactors compared with vesicular forms. Specifically, in nanoblends, poly-PCDA can engage in both poly-PCDA–poly-PCDA and poly-PCDA–P123 interactions. The latter competes with the interactions between adjacent PCDA monomers. It is noteworthy that the P123 copolymer, being the most hydrophobic among the investigated copolymers, forms stronger interactions with PCDA monomers and promotes a more hydrophobic environment for the insertion of PCDA monomers. This leads to increased distance between neighboring PCDA monomers and weakened PCDA–PCDA interactions. Consequently, diminished van der Waals forces between proximate carbon chains reduce the thermal energy required to disrupt poly-PCDA–poly-PCDA interactions and overcome the poly-PCDA rotational energy barrier, thereby facilitating the chromatic transition in nanoblends compared with vesicles.

Between PCDA and TCDA monomers, one might expect the poly-TCDA–P123 nanoblend to demonstrate a lower transition temperature compared with the poly-PCDA–P123 nanoblend. This expectation arises from the shorter carbon chain in poly-TCDA, which would result in fewer van der Waals interactions between the poly-TCDA tail and the hydrophobic region of the P123 copolymer, as well as less intense poly-TCDA–poly-TCDA. However, experimental observations do not support this expectation. This discrepancy suggests that hydrophobicity alone does not solely dictate the chromatic transition temperature of the nanostructures. Other factors, such as hydrophilicity and M_n_ of the copolymer, should also be taken into account. Consequently, it is proposed that the poly-PCDA–P123 nanoblend exhibit a lower color transition temperature due to the weakened poly-PCDA–poly-PCDA interactions. This weakening is attributed to the increased number of poly-PCDA–P123 interactions formed in the hydrophobic environment.

## 8. Applications of PDAs

PDAs exhibit a unique ability to change color in response to various external factors, such as temperature, pH, and the presence of specific chemical compounds. Notably, when exposed to rising temperature, they can act as reliable indicators, signaling potential breaches in temperature-sensitive environments like the cold supply chain. This capability is particularly critical in fields where temperature control directly influences product quality and safety, such as the handling of food, pharmaceuticals, and vaccines, which may degrade or become harmful under excessive heat.

Given this property, PDAs could also be effectively utilized in thermochromic indicators for tissue biobanking, which involves the collection, analysis, storage, and distribution of biospecimens for basic, translational, and clinical research [65]. By providing visual indications of temperature fluctuations in each stage, these indicators could enhance the monitoring and management of biological samples. Consequently, this would ensure their quality, integrity, safety, and viability, which are essential to obtaining reliable and reproducible results in genomic, transcriptomic, and proteomic assays [66,67].

However, one significant challenge during sample collection is the unrecorded time delay between patient excision and subsequent pathological or molecular analysis. This delay can lead to substantial alterations in protein signaling pathways within just 30 min of excision, triggering a stress response in the tissue [68]. During this response, cytokines are released due to wounding, resulting in hypoxia-related changes that can compromise biospecimen quality [69]. Both warm and cold ischemia can exacerbate these effects, negatively impacting the sample’s integrity and hindering subsequent analysis [70]. Moreover, prolonged hypoxia further deteriorates biospecimen quality by increasing tissue lactate levels and lowering pH. Microarray studies suggest that the extent of acidosis during the agonal state could be a key factor influencing RNA variation across biospecimens [71,72,73].

Once autopsy samples are procured, it becomes imperative to keep them on ice, and all handling procedures must be carried out promptly to avoid degradation. PDA-based thermochromic sensors could play a pivotal role in this process by providing immediate visual alerts when temperature or time thresholds are exceeded. This feature helps prevent mishandling and ensures that samples remain intact prior to freezing and storage. For long-term preservation, tissues, blood, urine, and other biospecimens that require high nucleic acid or protein integrity must be maintained at ultralow temperature, typically −80 °C or lower [72,74,75,76]. While maintaining high-quality biosamples is crucial for biomedical research, repeated freeze–thaw cycles during storage can degrade biomolecule stability. In fact, research by Ji et al. demonstrated that RNA integrity is better preserved when thawed on ice rather than at room temperature [77].

Integrating these thermochromic sensors into storage protocols could facilitate real-time temperature monitoring, provide immediate visual alerts for any deviations, and simplify compliance with audit requirements. Furthermore, during the transportation of biobanked samples, maintaining controlled conditions is critical. Here, these sensors could offer a reliable method to track temperature variations, ensuring the reliability of the biobank.

In addition to temperature fluctuations, PDAs can also alter color in response to shifts in pH levels that occur during spoilage, as well as to the volatile chemical compounds released during this process [78]. This versatility allows PDAs not only to serve as temperature indicators but also as freshness sensors for food products such as milk, meat, and fish. Some of these sensors can even function at sub-ambient temperature, which is particularly beneficial for monitoring the spoilage of temperature-sensitive goods. Therefore, this review specifically focuses on them.

For instance, Weston et al. created a PDA/ZnO/agarose disc designed to detect milk quality from 4 °C to room temperature [79]. As milk spoils, bacteria convert lactose into lactic acid, resulting in a pH reduction from 6.8 to 4.0. The increasing acidity leads to ZnO dissociation, disrupting interactions with PDA and causing a visible color change. By selecting the appropriate DA monomer and adjusting lipid doping, the system’s sensitivity to lactic acid can be finely tuned. Consequently, it can distinguish between fresh (pH 6.8–6.0), spoiling (pH 6.0–4.5), and spoiled milk (pH 4.5–4.0), which is indicated by a corresponding blue-to-purple-to-red color shift.

In another example, Nguyen et al. developed a PDA/cellulose nanocrystal/chitosan film tailored for detecting ammonia emissions from spoiling meat, operating from −20 °C to room temperature [80]. As meat spoils, proteolysis leads to an increase in ammonia levels, which this innovative sensor can monitor with high sensitivity. Notably, the film demonstrates a visible color change with 500 ppm ammonia under sub-zero conditions and detects 100 ppm at room temperature, providing a reliable indicator of product quality. This capability allows for the identification of spoilage before it becomes visually apparent.

In addition to meat, Li et al. fabricated a PDA/DMPC/cellulose nitrate membrane to detect fish spoilage between −20 °C and room temperature by measuring histamine levels, which increase as fish ages [81]. The sensor is stable for two weeks at room temperature and exhibits a linear response to histamine concentrations ranging from 70 to 2240 ppm, with minimal interference from gases such as nitrogen, oxygen, carbon dioxide, argon, and hydrogen sulfide. This specificity makes it a highly reliable tool for monitoring fish freshness over extended periods.

By incorporating these PDA-based indicators into food packaging, stakeholders can obtain immediate visual feedback on the freshness and safety of perishable goods. This proactive approach not only ensures that products meet quality standards before consumption but also significantly enhances food safety while reducing waste.

## 9. Future Directions

PDAs, which exhibit irreversible color changes at sub-ambient temperature, present exciting opportunities for precise temperature monitoring in various industries. The future directions outlined here pave the way for a deeper understanding and broader exploration of these materials.

One particularly promising avenue to advance this understanding is the use of computational modeling and simulation techniques. The application of advanced methods, such as molecular dynamics simulations and density functional theory (DFT), provides an opportunity to predict the behavior of novel DA monomers. This predictive capability enables researchers to investigate the relationships between molecular structures and the thermochromic properties of PDAs. By applying this knowledge, suitable materials for low-temperature applications can be identified more efficiently while simultaneously establishing a theoretical framework that will better guide future experimental efforts.

Building on these computational insights, the next significant step naturally shifts toward the strategic synthesis of DA derivatives. By designing side chains and functional groups that modulate intermolecular interactions between DA monomers, researchers can further refine the properties of PDAs. This systematic exploration will not lead to the development of materials with precisely controlled thermochromic transition temperature but also deepen our understanding of how specific molecular modifications influence thermochromic behavior. With this knowledge, predictive models can be constructed to tailor PDAs to specific temperature ranges in a targeted manner.

As research progresses, blending synthesized DA derivatives with copolymers represents another promising direction. This strategy could yield materials that can be rapidly evaluated by using high-throughput screening platforms. Advanced analytical methods, such as ultraviolet differential scanning calorimetry (UV-DSC) and temperature-dependent UV–vis spectroscopy, will play a crucial role in assessing these materials. Such rapid assessments will accelerate the identification of compositions with desired thermochromic properties, which, in turn, will facilitate the development of PDAs designed for specific industrial applications.

**Figure 2:**
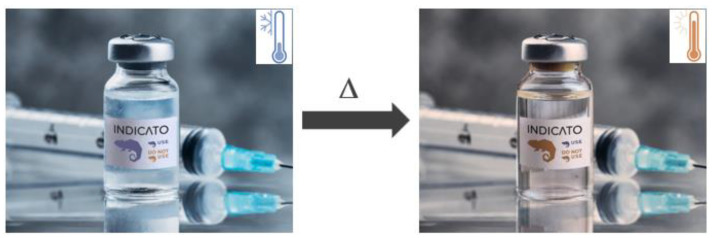
Example of using a PDA-based temperature indicator on a vaccine vial label (the idea of the figure was taken from [82]).

In practical terms, PDAs exhibiting thermochromic behavior at low temperature could serve an important role in industries where precise temperature control is required. They could be applied in the transportation of pharmaceuticals (Figure 2), the preservation of thermolabile medical products, and the monitoring of biobanking samples. By integrating PDAs into temperature-monitoring systems, the real-time tracking of temperature fluctuations becomes possible, ensuring that sensitive products remain within their optimal temperature ranges and thus maintaining their effectiveness and safety. To bring these innovations to the market, collaboration with industry partners will be essential. Such partnerships will help define specific functional requirements and support the advancement of PDAs for real-world applications.

However, to ensure the long-term success of PDAs in these applications, their environmental impact must be carefully considered. The sustainable development of these materials will require future research to prioritize the creation of non-toxic and environmentally friendly DA monomers. Comprehensive studies of cytotoxicity and migration will be necessary to confirm that PDAs are safe for human health and the environment. Moreover, evaluating the long-term stability and degradation behavior of these materials under various conditions will be crucial to verifying their sustainable in practical use.

In summary, the future directions provided here offer a roadmap for advancing the field of PDAs showing low-temperature thermochromism. The insights gained from the proposed approaches will not only enhance our understanding of these materials but also contribute to the development of innovative solutions with far-reaching impacts across different industries. By focusing on these research directions, PDAs can continue to evolve, opening new opportunities for temperature-sensitive applications.

## 10. Conclusions

This review highlights the burgeoning field of low-temperature thermochromism, emphasizing its importance and potential in diverse applications, particularly in the biobanking sector. Thermochromic materials, especially polydiacetylenes (PDAs), have shown significant promise due to their ability to provide visible, irreversible color changes at sub-ambient temperature. These materials are particularly advantageous for monitoring temperature-sensitive biological samples, ensuring their integrity throughout storage and transportation.

PDAs, with their unique ene-yne backbone structure, exhibit a range of color changes in response to temperature variations. Recent advances have demonstrated that manipulating the chemical structure of DA monomers, such as adjusting side-chain lengths and functional groups, can effectively tune the thermochromic transition temperature of PDAs (Table 2). Innovations like the use of copolymers further expand the potential of PDAs by enabling precise control over their thermal response and improving their applicability in real-world scenarios.

The integration of PDAs into biobanking practices represents a significant step forward in maintaining the quality of biological specimens. By offering real-time visual indications of temperature fluctuations, these thermochromic materials enhance the monitoring and management of biosamples, addressing critical issues such as temperature excursions during storage and transportation. This capability is essential to preserving the integrity of sensitive materials like biological samples, but also vaccines and pharmaceuticals, which are pivotal for research and medical applications.

Future research should focus on refining the synthesis of DA derivatives to achieve even lower thermochromic transition temperatures and better control over their properties. Computational modeling and high-throughput screening techniques will play crucial roles in accelerating the development of novel PDAs tailored for specific applications. Collaborative efforts between researchers and industry will be instrumental in translating these advances into practical solutions for temperature monitoring.

In summary, the ongoing developments in low-temperature thermochromism offer exciting opportunities to advance temperature-sensitive technologies. The ability to monitor temperature changes with high precision and reliability holds promise for improving the management of valuable biological materials and other thermolabile products, ultimately contributing to better outcomes in research, medicine, and beyond.

## Figures and Tables

**Table 1:** Summary of applications, types of thermochromic materials, and their mechanisms of action.

Application	Thermochromic Material	Mechanism	Reference
Invisible ink	Organic	Change in molecular structure	[7]
Heat-sensitive fax paper	Organic	Change in molecular structure	[8]
Hypercolor T-shirt	Organic	Change in molecular structure	[9]
Industrial heating device	Organic	Phase transition	[11]
Fire-resistant coating	Organic–inorganic	Change in coordination geometry and number	[12]
Monitoring of body temperature	Organic	Phase transition	[13]
Monitoring of blood circulation	Organic	Phase transition	[14]

**Table 2:** The influence of structural modifications of DA monomers on the thermochromic temperature of the corresponding PDAs.

Structural Parameter	Structural Modification	Thermochromic Temperature
Number of methylene units in side chain	Decrease	Decrease
Number of methylene units between DA moiety and any functional group	Decrease	Decrease
Number of hydrogen-bonding interactions	Decrease	Decrease
Number of PO segments in copolymer	Increase	Decrease
